# Impact of Newly Introduced Population-Based Screening and the COVID-19 Pandemic on Colorectal Cancer Incidence and Characteristics: A Retrospective Cohort Study

**DOI:** 10.14309/ctg.0000000000001004

**Published:** 2026-02-20

**Authors:** Renáta Bor, Béla Vasas, Zsófia Bősze, Anna Fábián, Mónika Szűcs, Dániel Magyar, Mariann Rutka, Tibor Tóth, Emese Ivány, Flóra Szántó, Anita Bálint, Bernadett Farkas, Péter Bacsur, Klaudia Farkas, Tamás Molnár, Zoltán Szepes

**Affiliations:** 1 Department of Internal Medicine, Center for Gastroenterology, Albert Szent-Györgyi Clinical Centre, University of Szeged, Szeged, Hungary;; 2 Department of Pathology, Albert Szent-Györgyi Clinical Centre, University of Szeged, Szeged, Hungary;; 3 Department of Medical Physics and Informatics, Albert Szent-Györgyi Clinical Centre, University of Szeged, Szeged, Hungary;; 4 HCEMM-USZ Translational Colorectal Research Group, Szeged, Hungary.

**Keywords:** COVID-19 pandemic, colorectal cancer, CRC screening, CRC epidemiology

## Abstract

**INTRODUCTION:**

In Hungary, population-based colorectal cancer (CRC) screening was introduced shortly before the onset of COVID-19 pandemic. We aimed to assess the combined impact of these 2 factors on the incidence and characteristics of CRC.

**METHODS:**

The retrospective cohort study included all patients with newly diagnosed CRC between 2014 and 2023 and divided into prepandemic with screening (2015–2016, 2019), prepandemic without screening (2014, 2017–2018), pandemic (2020–2021), and postpandemic (2022–2023) subgroups. CRC incidence, diagnostic patterns, and tumor stage were compared across subgroups.

**RESULTS:**

Crude CRC incidence in institution's care area was lower during pandemic (102.51 per 100,000) compared with the prepandemic with screening (117.92 per 100,000; *P* = 0.060) and postpandemic (120.60 per 100,000; *P* = 0.044) subgroups. Age-standardized incidence differed significantly only between the prepandemic with screening and pandemic subgroups (125.30 vs 105.88 per 100,000; *P* < 0.001). During pandemic, the proportion of early-stage (on the American Joint Committee on Cancer 0–I) cancers was significantly reduced compared with the prepandemic with screening subgroup (18.74% vs 25.08%; *P* = 0.048) and the proportion of T1 cancers was also lower compared with the postpandemic subgroup (6.90 vs 12.2%; *P* = 0.026). During pandemic, both the annual number of colonoscopies (2,706.00) and the mean number of colonoscopies required to detect 1 CRC (15.56) were markedly lower compared with the prepandemic with screening (4,590.67 and 13.97), prepandemic without screening (3,824.33 and 10.67), and postpandemic (4,573.50 and 15.50) subgroups.

**DISCUSSION:**

The COVID-19 pandemic was associated with unfavorable changes in CRC epidemiology. Organized screening may have mitigated the negative impact during the pandemic and postpandemic periods.

## Graphical abstract

**Figure:**
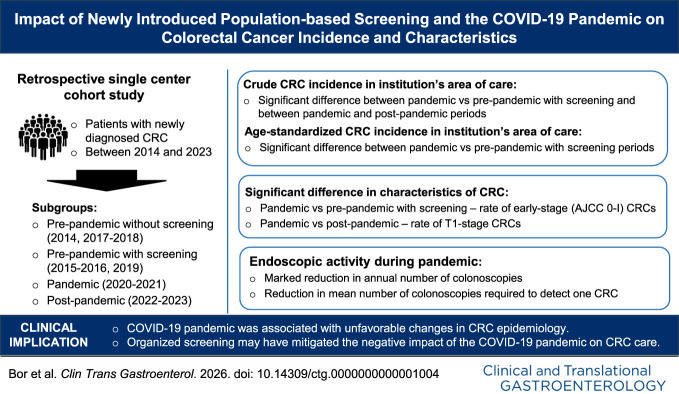

## INTRODUCTION

The COVID-19 pandemic has significantly affected gastroenterology care, necessitating the restructuring of workflows and protocols. In addition, restrictions on endoscopic procedures due to heightened risk of viral transmission have further limited patients' access to health care. Early in the pandemic, the European Society of Gastrointestinal Endoscopy (ESGE) published guidelines providing detailed recommendations for pre-endoscopy, intraendoscopy, and postendoscopy practices during the crisis, as well as a prioritization framework for endoscopic indications (1,2). Concurrently, most countries established local and national professional protocols. Most measures were implemented during the pandemic waves, followed by a compensatory increase in endoscopic examinations during the intervening periods. In Hungary, the overall number of upper and lower gastrointestinal endoscopies did not decrease in the first year of the COVID-19 pandemic (2020) compared with the reference year (2019). However, during peak phases of the pandemic, the volume of both upper and lower endoscopies dropped by 80% (3). During the pandemic waves, a decrease in the incidence of tumors, including colorectal cancer (CRC), was reported. This was accompanied by a higher rate of metastatic disease and emergency presentations, as well as the diagnostic delays that could contribute to tumor progression and to more advanced stage at diagnosis (4–7). The significance of CRC is highlighted by the fact that it is the third most common cancer worldwide, accounting for 9.3% of all cancer-related deaths (8). To enhance epidemiological data and early detection, many countries have implemented population-based, organized CRC screening programs targeting individuals at average risk. In Europe, substantial heterogeneity exists regarding target age ranges, population coverage, and screening modalities (9). Most national screening programs target average-risk individuals aged 50–70 years; however, several countries extend eligibility to younger populations aged 45–50 years (e.g., the Czech Republic, Austria, and Slovakia) or to older individuals older than 70 years of age (e.g., Austria, Sweden, and Slovakia). Stool-based fecal blood tests constitute the most used primary screening modality, although in some countries colonoscopy is also offered as a first-line screening option (including Austria, Greece, and Czech Republic) (10). In the United States, after 15 years of CRC screening and achieving an up-to-date screening rate above 80%, annual CRC incidence declined by more than 25%, while mortality was reduced by more than 50% (11). However, the COVID-19 pandemic has also disrupted screening programs in many countries, resulting in temporary suspensions in some and reduced capacity in others, accompanied by heightened precautionary measures. In Hungary, regional pilot CRC screening program was implemented in Csongrád-Csanád County from 2013, with colonoscopies predominantly performed in 2015–2016. Before this initiative, screening was conducted opportunistically. The population-based screening program was launched in 2018—just before the outbreak of the pandemic—by inviting individuals to undergo fecal immunochemical test (FIT), with screening colonoscopies beginning in 2019. The monthly number of colonoscopy examinations plateaued in early 2020. In line with ESGE guidelines, a positive FIT result in the screening program was considered a high-priority indication; therefore, colonoscopies were not postponed in these cases. However, overall endoscopic capacity was significantly reduced.

In Hungary, the simultaneous launch of the screening program and the onset of the pandemic created a unique situation because the years 2020 and 2021 were influenced by 2 opposing factors affecting CRC incidence and characteristics. The aim of our study was to assess the combined impact of the COVID-19 pandemic and the newly implemented CRC screening program on CRC incidence and clinical characteristics.

## METHODS

### Study design and patient enrollment

In this retrospective cohort study, we assessed the effects of both the COVID-19 pandemic and the Hungarian population-based colorectal screening program launched shortly before the outbreak on the detection and clinical characteristics of CRC between 2014 and 2023 in Csongrád-Csanád County. All patients diagnosed with CRC during the study period at the University of Szeged were enrolled in the study. We retrieved the list of patients diagnosed with International Classification of Diseases codes C18, C19, and C20 from the Department of Pathology's database to identify the affected individuals. After eliminating duplicates, the following cases were excluded from the study: (i) CRC cases identified at another institution where only specific oncological and/or surgical treatment was conducted at our center; (ii) recurrent CRCs; (iii) extracolonic tumors involving the colon; (iv) CRC was diagnosed out of study period; (v) despite the clinical suspicion of cancer, the colonoscopy, surgery, and/or pathological examination did not confirm the diagnosis of CRC.

In the anonymized database, we collected basic demographic information of patients (such as sex and age) and clinical characteristics of cancer, including tumor, node, metastasis (TNM) stage and method of diagnosis. Regarding the method of diagnosis, we distinguished lesions confirmed through clinical examination (colonoscopy or imaging if colonoscopy was not feasible or contraindicated) and those identified in conjunction with tumor-related complications (such as ileus, perforation, abdominal abscess, and intestinal necrosis). In addition, we determined the rate of interval postcolonoscopy CRC using the definition provided by the consensus statement of the World Endoscopy Organization: Any CRC detected before the subsequent recommended surveillance colonoscopy was categorized as interval cancer (12). The essential diagnostic and clinical indicators examined in this study are routinely and comprehensively recorded in our institution; therefore, no missing or incomplete data were present. Patient follow-up data were not collected or analyzed. Medical documentation of patients and cancers was collected using a MedSolutions medical recorder.

The primary objective of this study was to evaluate the combined impact of the COVID-19 pandemic and the implementation of a population-based 2-step CRC screening program, initiated immediately before the pandemic on the incidence and clinical characteristics of CRC. The secondary objective was to assess the impact of the pandemic on local gastrointestinal endoscopic care and to analyze the correlation between change in endoscopic care performance and the CRC occurrence.

### Characteristics of study period and determination of subgroups

To assess the effects of the pandemic and CRC screening program on the CRC detection and characteristics, the study cohort was divided into 3 subgroups: prepandemic, pandemic, and postpandemic.

**Figure 1.**
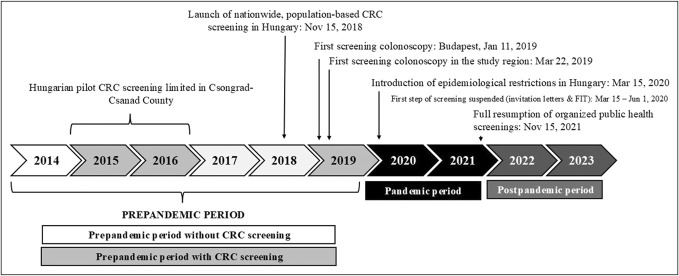
Timeline of the most important public health events during the study period and study subgroups. CRC, colorectal cancer; FIT, fecal immunochemical test.

The prepandemic subgroup included patients who were diagnosed with CRC between 2014 and 2019. A longer prepandemic interval was required for comparison with the pandemic and postpandemic periods because CRC screening influenced this timeframe only partially and intermittently. The Hungarian pilot CRC screening, limited in Csongrád-Csanád County, was performed in 2015 and 2016, which may have influenced the incidence of CRC and potentially affected the stage of diagnosed cancers. The 2-step population-based CRC screening program was launched in 2018 with distribution of invitation letters for FIT; however, screening colonoscopies for individuals with positive FIT results started only in the second quarter of 2019. Therefore, in 2018, screening activities had no epidemiological impact on CRC. Based on these considerations, we further divided this subgroup into 2 parts, a prepandemic period with screening (years 2015, 2016, and 2019) and a prepandemic period without screening (years 2014, 2017, and 2018) (Figure 1).

The pandemic period encompassed patients diagnosed with CRC in 2020 and 2021, while the postpandemic period included cases confirmed in 2022 and 2023. Owing to the COVID-19 pandemic in Hungary, nonurgent medical interventions were postponed from March 11, 2020, to May 4, 2020, and again from November 5, 2020, to April 29, 2021. During the interim period, although healthcare services had resumed, endoscopic procedures were subject to special regulations due to their heightened risk of virus transmission. In addition, the reallocation of healthcare personnel and temporary closure of several endoscopy units further limited the capacity for care. The prepandemic operations of healthcare facilities gradually resumed in the latter half of 2021 (3). In Hungary, the 2-step population-based CRC screening program was launched in 2018 with distribution of invitation letters for FIT, and in the second half of 2019, screening colonoscopies started performing for individuals with positive FIT results. During the pandemic period, the distribution of invitation letters and the administration of FIT were halted from March 15, 2020, to June 1, 2020. According to the ESGE and the Hungarian professional position statement, endoscopy is considered a high-priority procedure for patients with positive FIT results within the organized screening program (1,2). Consequently, depending on institutional capacity, the colonoscopy was conducted with heightened precautions (only in low-risk patients with negative COVID-19 rapid test, using appropriate protective equipment). From June 2020, both components of the screening process, the FIT determinations and endoscopic examinations, were resumed with strict precautions: All patients underwent rapid COVID-19 testing just before their colonoscopy; furthermore, negative polymerase chain reaction (PCR) result required from patients vaccinated more than 4 months ago. These protocols were only rescinded on 7 March 2022.

In the 4 subgroups generated, we compared the absolute number of newly confirmed CRCs, the crude and age-standardized incidence of CRC in the care area covered by our institution, the TNM stage distribution of cancers, and the proportion of tumor stages according to the 8th edition of the American Joint Committee on Cancer (AJCC). Based on this classification, 4-stage categories were used: early (stage 0 and I), localized (stage II), locally advanced (stage III), and metastatic (stage IV). The CRC incidence in the care area of the institution does not correspond to the real CRC incidence in Csongrád-Csanád County because the calculation is based on the number of residents in the area of care, while our tertiary level referral medical center also receives a large number of patients from other parts of the country. However, the use of this indicator can illustrate the changes in health care services in the context of the COVID-19 pandemic. Furthermore, we examined potential discrepancies in the rate of CRC cases confirmed in complicated forms and the occurrence of interval CRCs across the subgroups. The number of endoscopic examinations performed and their indication could potentially influence the incidence of CRC, so we examined potential disparities in the absolute number of colonoscopies across subgroups. The quantity of examinations performed was determined by the number of screening and conventional colonoscopic examination funding codes reported to the health insurer. In addition, within each subgroup, we calculated the average number of colonoscopies required to detect a CRC.

### Ethics approval and consent to participate

The study was approved by the National Scientific and Ethical Committee of Hungarian Medical Research Council (ETT TUKEB; ethics approval number: TUKEB: BM/3496-1/2023). All the included patients have signed an informed consent form for the scientific use of their medical data. The study was performed in accordance with the Declaration of Helsinki (13).

### Statistical analysis

Statistical analysis was performed with R statistical software version 4.2.0 (R Foundation); *P* values of less than 0.05 were considered significant. Descriptive statistics are presented as mean ± SD for continuous variables and as absolute numbers and percentages for categorical variables. CRC incidence in the institution's care area was calculated using both crude incidence rates and age-standardized incidence rates. Age standardization was performed using the age structure of the population covered by the University of Szeged as the reference population, to account for temporal changes in population size and age structure during the study period. Comparisons of incidence rates, as well as clinical and stage-related tumor characteristics across the predefined study periods, were conducted using Pearson χ^2^ tests. Pairwise comparisons between individual study periods were performed, and *P* values were adjusted using the Holm correction method to control for multiple testing. Given the retrospective design and the inclusion of the entire eligible population, a formal sample size estimation was not performed. The reporting of this study conforms to the Strengthening the Reporting of Observational Studies in Epidemiology (STROBE) statement (14).

## RESULTS

### Characteristics of study cohort

During the study period, a total of 2,868 tumors were identified in 2,803 patients. Among these patients, synchronous CRC was confirmed in 58 cases. The male sex represented a higher proportion (58.65%) compared with women in the cohort. The mean age of patients at diagnosis was 68.51 ± 11.43 years (ranging from 21 to 97 years, median 69 years). Left colon cancer accounted for 73.81% of all CRC cases. A total of 265 CRC cases were diagnosed at the complication stage. Urgent surgery confirmed the presence of a tumor in the setting of subileus/ileus in 209 cases and bowel perforation in 43 cases. In addition, 3 cases were identified during fistula surgery and another 3 during abdominal abscess exploration. CRC was also diagnosed in the context of appendectomy (n = 2), acute mesenteric ischemia (n = 1), extensive intestinal necrosis (n = 1), and gastrointestinal hemorrhage investigation (n = 2), with 1 case confirmed only with autopsy. Further details regarding the distribution of cancers within the colon are provided in Tables 1 and 2. There were no substantial differences between subgroups in gender distribution, mean age at diagnosis, and tumor location.

**Table 1.** Clinical characteristics of study population and colorectal cancers

	Prepandemic with screening (2015, 2016, 2019)	Prepandemic without screening (2014, 2017, 2018)	Pandemic (2020–2021)	Postpandemic (2022–2023)
Total number of CRC	885	821	507	590
Annual number of CRC cases	295.00	273.67	253.50	295.00
Male sex	527 (59.55%)	498 (60.66%)	291 (57.40%)	328 (55.59%)
Age at the diagnosis	68.08 ± 10.79	68.23 ± 12.02	68.49 ± 11.78	69.56 ± 11.16
Synchronous CRC	16 (1.81%)	23 (2.80%)	12 (2.37%)	9 (1.53%)
Cancer location				
Anorectum	312 (34.78%)	294 (34.83%)	192 (36.78%)	221 (36.89%)
Sigmoid colon	273 (30.17%)	266 (31.52%)	141 (27.01%)	163 (27.21%)
Descending colon	42 (4.64%)	15 (1.78%)	18 (3.45%)	18 (3.01%)
Splenic flexure	17 (18.78%)	31 (3.67%)	25 (4.75%)	27 (4.51%)
Transverse colon	39 (4.31%)	39 (4.62%)	27 (5.17%)	28 (4.67%)
Hepatic flexure	49 (5.41%)	52 (6.16%)	25 (4.75%)	37 (6.18%)
Ascending colon	91 (10.06%)	70 (8.29%)	42 (8.05%)	41 (6.84%)
Cecum	82 (9.06%)	77 (9.12%)	52 (9.96%)	64 (10.68%)
Interval CRC	34 (3.84%)	46 (5.60%)	13 (2.56%)	21 (3.56%)

CRC, colorectal cancer.

**Table 2.** Staging characteristics of colorectal cancers based on TNM and AJCC classification in the study population

	Prepandemic with screening (2015, 2016, 2019)	Prepandemic without screening (2014, 2017, 2018)	Pandemic (2020–2021)	Postpandemic (2022–2023)
T stage				
Tis	31 (3.50%)	22 (2.68%)	4 (0.79%)	3 (0.51%)
T1	93 (10.51%)	78 (9.50%)	35 (6.90%)	72 (12.20%)
T2	135 (15.25%)	116 (14.13%)	78 (15.38%)	84 (14.24%)
T3	438 (49.49%)	458 (55.79%)	254 (50.10%)	298 (50.51%)
T4	188 (21.24%)	147 (17.90%)	136 (26.82%)	133 (22.54%)
Lymph node metastasis (≥N1)	406 (45.88%)	393 (47.87%)	259 (51.08%)	281 (47.63%)
Distant metastasis (M1)	219 (24.75%)	232 (28.26%)	122 (24.06%)	134 (22.71%)
AJCC stage				
0	31 (3.50%)	22 (2.68%)	4 (0.79%)	3 (0.51%)
I	191 (21.58%)	159 (19.37%)	91 (17.95%)	142 (24.07%)
II	227 (25.65%)	200 (24.36%)	135 (26.63%)	146 (24.75%)
III	217 (24.52%)	208 (25.33%)	155 (30.57%)	165 (27.97%)
IV	219 (24.75%)	232 (28.26%)	122 (24.06%)	134 (22.71%)
CRC detected at complication stage	77 (8.70%)	70 (8.77%)	61 (12.03%)	57 (9.66%)

AJCC, American Joint Committee on Cancer; CRC, colorectal cancer; TNM, tumor, node, metastasis.

### Trends in CRC incidence and clinical characteristics

**Figure 2.**
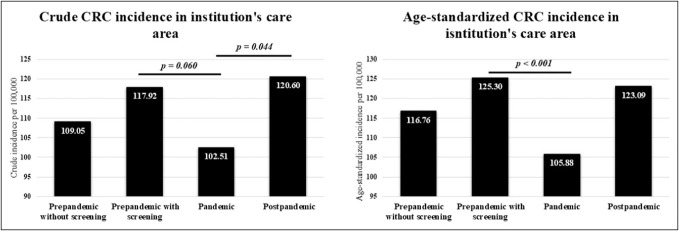
Comparison of crude and age-standardized CRC incidence rates in the institution's care area across the 4 study subgroups. CRC, colorectal cancer.

**Figure 3.**
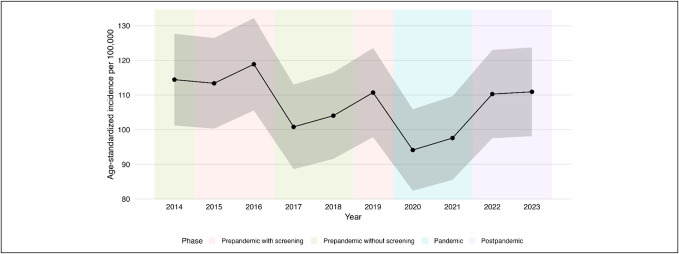
Age-standardized colorectal cancer incidence in the institution's area of care over the 10-year study period.

In the pandemic subgroup, an average of 253.50 CRCs were diagnosed per year, which was substantially lower than in the prepandemic with screening (295.00 cases/yr), prepandemic without screening (273.57 cases/yr), and postpandemic (295.00 cases/yr) subgroups. The crude incidence rate of CRC calculated for the institution's area of care in the pandemic subgroup (102.51 per 100,000) was significantly lower than in both the prepandemic with screening (117.92 per 100.000; *P* = 0.060) and postpandemic (120.60 per 100,000; *P* = 0.044) subgroups (Figure 2). After age standardization, a significant difference in the incidence rate of CRC calculated for the institution's area of care was observed only between the prepandemic with screening and pandemic subgroups (125.30 vs 105.88 per 100,000; *P* < 0.001) (Figure 3). Although the prepandemic without screening subgroup showed a higher crude CRC incidence (109.05 per 100,000; *P* = 0.548) than the pandemic subgroup, it remained lower than in the prepandemic with screening and postpandemic subgroups; however, these differences were not statistically significant (*P* = 0.319 and *P* = 0.249, respectively).

**Figure 4.**
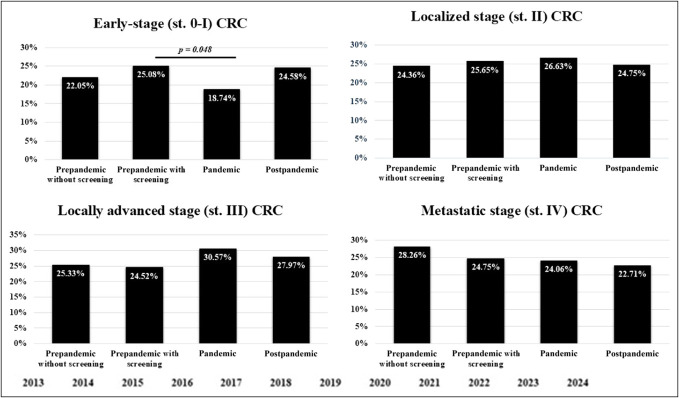
Comparison of AJCC (8th edition) tumor stage distribution across the 4 study subgroups. A significant difference was observed only in the proportion of early-stage tumors (AJCC stages 0–I) between the pandemic and prepandemic with screening subgroups. AJCC, American Joint Committee on Cancer; CRC, colorectal cancer.

When examining the prevalence of pT1 stage tumors, a significant difference was found only between the pandemic and postpandemic subgroups (6.90% vs 12.20%, *P* = 0.026). In parallel, the proportion of early-stage (AJCC stage 0-I) tumors was the lowest in the pandemic subgroup compared with the prepandemic with screening, prepandemic without screening, and postpandemic groups (18.74% vs 25.08% vs 22.05% vs 24.58%, respectively), but the difference was only significant when compared with the prepandemic with screening (Figure 4). It is noteworthy that there has been a consistent decrease in the proportion of Tis tumors over the years, irrespective of CRC screening and the COVID-19 pandemic (4.18% in 2014, 10.38% in 2015, 0.10% in 2016, 2.68% in 2017, 1.11% in 2018, 0.69% in 2019, 1.2% in 2020, 0.39% in 2021, 1.02% in 2022, and 0% in 2023). The pandemic subgroup did not differ statistically from all 3 groups in the proportion of localized stage (26.63% vs 25.65% vs 24.36% vs 24.75%) and locally advanced stage (30.57% vs 24.65% vs 25.33% vs 27.97%) tumors. At the time of diagnosis, the proportion of metastatic-stage (AJCC stage IV) tumors was highest in the prepandemic with screening subgroup (28.26%) and lowest in the postpandemic subgroup (22.71%). The absolute number and proportion of CRC cases diagnosed at the complication stage were highest in the pandemic subgroup (30.50 cases/yr), compared with the prepandemic with screening (25.67 cases/yr), prepandemic without screening (23.33 cases/yr), and postpandemic (28.50 cases/yr) subgroups; however, this difference was not statistically significant (*P* = 0.146). The incidence rate of postcolonoscopy CRC in the study cohort was 4.07%, with the lowest rate observed in the pandemic subgroup (2.56%) and the highest in the prepandemic with screening subgroup (5.60%). This difference was not statistically significant (*P* = 0.013).

Examining the impact of the screening program, we found that in the extended prepandemic cohort, only minor, statistically nonsignificant differences were observed between the subgroups with and without CRC screening regarding the proportion of T1 stage (10.51% vs 9.50%, *P* = 0.707), early-stage (25.08% vs 22.05%, *P* = 0.156), localized stage (25.65% vs 24.36%, *P* = 1.000), locally advanced stage (24.52% vs 25.33%, *P* = 0.889), and metastatic (24.75% vs 28.10%, *P* = 0.531) tumors, as well as CRC diagnosed at complication stage (3.84% vs 5.60%, *P* = 0.109) and interval postcolonoscopy CRCs (8.70% vs 8.77%, *P* = 1.000). The closest similarity in the clinical characteristics of cancer was found between the prepandemic with screening and postpandemic subgroups, and there were no statistically significant differences between these subgroups regarding any of the factors discussed above.

### Association between endoscopic care performance and CRC incidence

A total of 39,804 complete colonoscopies were performed during the study period, averaging 3,980.40 procedures per year and 14.20 colonoscopies per detected CRC case. However, significant differences were observed between the study subgroups in this metric. The annual number of colonoscopies increased by 20.04% in the prepandemic with screening subgroup (4,590.67 per year) compared with the prepandemic without screening subgroup (3,824.33 per year). The absolute number of procedures in the prepandemic with screening period was nearly identical to that of the postpandemic period, which was also influenced by screening (4,573.50 per year). Within the extended prepandemic group, there was no relevant difference between the 2 subgroups in the mean number of colonoscopies required to detect 1 CRC (15.56 vs 13.97), and it showed high similarity with postpandemic period (15.50).

**Figure 5.**
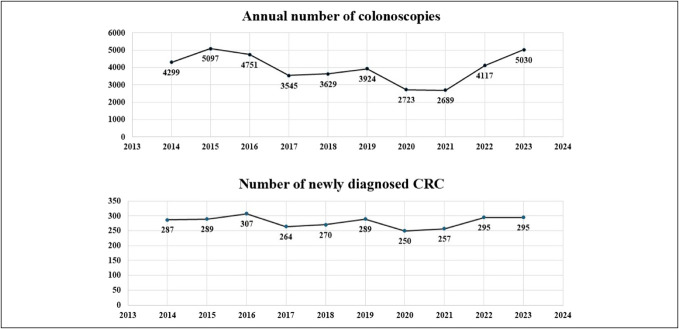
Trends in the annual numbers of newly diagnosed colorectal cancers and colonoscopies during the study period. CRC, colorectal cancer.

During the pandemic period, the annual number of colonoscopies (n = 2,706) decreased a substantial decline of 64.31% in comparison with the prepandemic annual figures (n = 4,208) (Table 3). Although the annual number of colonoscopies in the postpandemic subgroup represents a 69.01% increase compared with the pandemic period, it merely returns to the level observed during the prepandemic with screening period and reflects only a 19.59% increase relative to the prepandemic without screening subgroup, so offering only partial compensation for the shortage experienced during the pandemic. Although detailed registry data on colonoscopy indications were unavailable, legal restrictions introduced during the pandemic likely led to changes in referral patterns. This is further supported by the increased diagnostic yield during the pandemic: On average, 1 CRC was detected per 10.67 colonoscopies, compared with 13.97, 15.56, and 15.50 in the prepandemic without screening, prepandemic with screening, and postpandemic periods, respectively (Figure 5).

**Table 3.** Association between endoscopic care performance and CRC incidence

	Prepandemic with screening (2015, 2016, 2019)	Prepandemic without screening (2014, 2017, 2018)	Pandemic (2020–2021)	Postpandemic (2022–2023)
Total number of colonoscopies	13,772	11,473	5,412	9,147
Average number of colonoscopies per year	4,590.67	3,824.33	2,706.00	4,573.50
Average number of patients underwent colonoscopy per year	4,193.00	3,510.00	2,570.00	4,172.00
Average number of colonoscopies required to detect 1 CRC	15.56	13.97	10.67	15.50

CRC, colorectal cancer.

## DISCUSSION

The results of our retrospective cohort study demonstrated that the COVID-19 pandemic had a significant negative impact not only on the number of newly diagnosed CRCs, but also on their clinical presentation. During the pandemic, our cohort showed a marked decrease in the proportion of early-stage (AJCC stage 0 and I) tumors compared with the prepandemic period, accompanied by an increase in CRC postcolonoscopy CRCs rate. These epidemiological changes in CRC incidence and clinical presentation were associated with a decrease in the number of colonoscopies performed during the pandemic. There was a surprisingly beneficial shift in CRC clinical characteristics, TNM and AJCC stage in the postpandemic period, with significantly increased proportion of T1 tumors, as well as elevation was observed in the proportion of early-stage and localized stage CRCs and decrease in the rates of locally advanced stage and metastatic stage CRCs; however, these differences did not reach statistical significance. The background for this positive change is assumed to be the initiation of population-based CRC screening, which uniquely in Hungary, was launched immediately before the onset of the COVID-19 pandemic and reached its target capacity based on colonoscopy numbers in the 3 months before the pandemic. The advantage of our study is that it examines the impact of these 2 events with opposite effects on the incidence and characteristics of CRC in a unique way. In addition, it demonstrates the potential ramifications of implementing noninvasive patient prescreening methods, such as FIT, during future epidemics. Hence, it can provide a reference point for the optimalization of work schedules and healthcare organization.

Consistent with our findings, Howlader et al, in their analysis of US cancer statistics, reported a statistically significant decline in incidence rates across all cancer types during the pandemic. This decrease was most pronounced for colorectal tumors (54.1%), among others, and was primarily observed in early-stage rather than late-stage disease (4). A retrospective multicenter cohort study from Italy, which analyzed clinical data of all patients who underwent surgery for CRC, also identified a significant association between the COVID-19 pandemic and an increased risk of more advanced oncologic stage at diagnosis (OR 1.07; 95% CI, 1.01–1.13; *P* = 0.03), more aggressive tumor biology (OR 1.32; 95% CI, 1.15–1.53; *P* < 0.001), and a higher incidence of stenotic lesions (OR 1.15; 95% CI, 1.01–1.31; *P* = 0.03) (7). In the Netherlands, the largest decline in the overall incidence of CRC occurred between April and June 2020, primarily, in 48% of cases driven the reduction in stage I tumors. By contrast, only 5% of the overall decline was attributable to a decrease in stage IV cases. From October 2020 onward, CRC case volumes began to rise and eventually surpassed the expected levels based on previous years. Since the decline was largely confined to stage I cancer, the authors attributed this trend to the temporary suspension of the national CRC screening program during the COVID-19 pandemic (15). Using 4 independent microsimulation models (ASCCA, MISCAN-Colon, OncoSim, and Policy1-Bowel), the study of Worthington et al estimated the long-term global impact of COVID-19-related disruptions to CRC screening. It projected approximately 13,000 additional CRC cases and 7,900 related deaths between 2020 and 2050. However, the appropriately implemented catch-up screening could have reduced the burden by more than 80% (16). Clearing the screening backlog in 24 months by modestly increasing the FIT threshold (by approximately 12.5%) could prevent the majority of excess CRC-related deaths resulting from a 3-month disruption while requiring only a minimal increase in colonoscopy demand (17).

A retrospective study from Spain also reported a reduction in the number of CRC cases, along with a higher proportion of tumors diagnosed at more advanced stages: positive lymph nodes in 52.3% vs 36.7% (*P* = 0.002) and metastatic disease in 23.6% vs 16.6% (*P* = 0.087). These findings were partly attributed to the suspension of CRC screening during the pandemic. The study also observed significantly longer diagnostic and treatment delays during the pandemic period: 6.4 months (95% CI, 5.8–6.9) compared with 4.8 months (95% CI, 4.3–5.3), *P* < 0.001 (18). The most significant delay occurred between the onset of symptoms and the establishment of a diagnosis. This delay may have been influenced by multiple factors, both patient-related (e.g., fear of infection, mandatory COVID-19 testing, and/or vaccination before endoscopic procedures) and system-related (e.g., reduced healthcare capacity, revised endoscopy indications, and suspension of CRC screening). Data on staging investigations after CRC diagnosis, as well as on the timing and delays of oncological and surgical treatment, vary across countries and different phases of the pandemic (19–22). Notably, during the COVID-19 pandemic, the number of average-risk patients undergoing their first colonoscopy declined significantly, with an even greater reduction observed among individuals from highly vulnerable communities (Social Vulnerability Index [SVI] > 0.8). Overall participation in screening and surveillance also decreased within these populations (23).

Population-based CRC screening was introduced in Hungary in 2018, with the first screening colonoscopies performed in 2019. Screening activity peaked at the end of 2019 and in the first quarter of 2020, followed by a plateau of approximately 700 screening colonoscopies per month. However, the distribution of FIT invitation letters was temporarily suspended during the first peak of the pandemic, contributed to a sharp decline in screening colonoscopies in 2021—down to one-fifth of previous levels. Volumes rebounded in 2022, returning to the earlier plateau phase through compensatory efforts. The operation of the screening program in the catchment area of our institution is summarized in Table 4, using data provided by the Hungarian National Centre for Public Health and Pharmacy (NNGYK). The results also indicate a substantial decline in the number of invitation letters distributed during the COVID-19 pandemic, followed by a marked increase in 2022. Concurrently, a decrease in the FIT return rate was observed. The pattern of change in screening colonoscopy numbers in our institution contrasts with that seen in most countries, where the decline in screening activity was predominantly observed in 2020 (24–26). The pronounced minimum in the annual number of screening colonoscopies was reached in 2021, with only 41 examinations performed. This represents a substantial decrease compared with 2019 (n = 241) and 2020 (n = 281), followed by a marked increase in subsequent years (2022: n = 625; 2023: n = 403). It should be emphasized that, within the national screening program, colonoscopies are restricted to 55 endoscopy units operating in 50 accredited institutions in Hungary. Consequently, patient recruitment was not confined to institutional catchment areas, so the screened population cannot be considered geographically restricted to the catchment areas of individual institutions. Consistent with the current screening program, patient referral during the pilot screening (2015–2016) was also independent of institutional catchment areas; during its entire duration, 503 screening colonoscopies were conducted at our institution (27). The exceptionally high incidence and mortality rates of CRC in Hungary have made the implementation of an organized, population-based screening program a public health priority, with the aim of achieving and sustaining adequate, up-to-date screening coverage in the population as early as possible. Over the longer term, this may enable the program to contribute to favorable changes in CRC epidemiological indicators after more than 15 years of operation.

**Table 4.** Operational characteristics of the population-based CRC screening program in the catchment area of our institution based on data from the registry of the Hungarian National Centre for Public Health and Pharmacy (NNGYK)

	Invitation letters sent (n)	FIT samples returned (n, %)	FIT-positive results (n, %)	Screening colonoscopy (n, %)
2018	2,960	1,123 (37.94%)	105 (9.35%)	71 (67.62%)
2019	13,957	5,381 (38.55%)	442 (8.21%)	279 (63.12%)
2020	7,656	2,002 (26.15%)	183 (9.14%)	115 (62.84%)
2021	1,331	282 (21.19%)	26 (9.22%)	15 (57.69%)
2022	38,823	6,954 (17.91%)	715 (10.28%)	501 (70.07%)
2023	3,775	404 (10.70%)	56 (13.86%)	35 (62.50%)
Total	68,502	16,146 (23.57%)	1,527 (9.46%)	1,016 (66.54%)

Annual data are categorized according to the year of invitation letter generation. The numbers and proportions of returned FIT samples, positive FIT results, and colonoscopies represent the outcomes of invitation letters generated in the respective year. However, in a substantial proportion of cases, FIT testing and subsequent colonoscopy were actually performed in the following calendar year.

CRC, colorectal cancer; FIT, fecal immunochemical test.

Consistent with international literature, our institution experienced a substantial 64.31% decrease in the number of colonoscopies during the pandemic compared with the prepandemic period (28). However, there was no significant difference in procedure volume between 2020 and 2021. Although specific data on colonoscopy indications are unavailable, a shift in indications is likely, as our institution adhered strictly to ESGE and national professional guidelines throughout the pandemic (1,2). This is supported by the markedly lower number of colonoscopies required to detect 1 case of CRC during the pandemic period compared with the prepandemic with screening, prepandemic without screening, and postpandemic periods (10.67 vs 15.56 vs 13.97 vs 15.50, respectively).

The main limitation of our study is that it is based on its reliance on localized data from Csongrád-Csanád County, rather than a comprehensive national analysis. Detailed data pertaining to both the pilot screening and the subsequent population-based national screening program were not available by region. This includes crucial metrics of first step of screening such as the number of invitations issued, patient adherence rate, rates of nonnegative FIT results, and the quality indicators of second step of screening such as colonoscopy acceptance rates, the total number of screening colonoscopies conducted, and rates of nonnegative examinations, as well as adenoma, polyp, and CRC detection rates. During the determination of the average number of colonoscopies required to detect 1 CRC, it was not possible to account for changes in population risk, age structure, or referral pathways because patient referrals often did not follow regional care regulations. This was partly due to our institution being a tertiary-level center and partly because screening colonoscopies were restricted to designated, accredited institutions. In addition, multiple comparisons across the 4 study subgroups may have introduced residual statistical uncertainty despite adjustment using the Holm-Bonferroni correction. Given the retrospective design and the long study period, unmeasured confounding related to temporal changes in population characteristics and referral practices cannot be entirely excluded.

In conclusion, our study observed unfavorable changes in CRC epidemiology during the COVID-19 pandemic, accompanied by a decrease in the number of colonoscopies performed. The newly introduced population-based CRC screening did not fully offset these changes during pandemic period; however, more favorable clinical tumor characteristics were noted in the postpandemic period coinciding with screening implementation. These findings suggest a potential role of organized screening in mitigating pandemic-related disruptions, although direct causal inference cannot be established. Furthermore, our results indicate that the use of FIT as a prescreening tool for asymptomatic individuals may represent a feasible noninvasive approach to support CRC detection during periods of healthcare system strain.

## CONFLICTS OF INTEREST

**Guarantor of the article:** Renáta Bor, MD, PhD.

**Specific author contributions:** R.B.: writing—original draft, methodology, investigation, data curation, conceptualization. B.V.: investigation, supervision, writing—review and editing. Z.B.: investigation, writing—review and editing. A.F.: investigation, writing—review and editing. M.S.: formal analysis. D.M.: formal analysis. M.R.: investigation. T.T.: investigation. E.I.: investigation. F.S.: investigation. A.B.: investigation. B.F.: investigation. P.B.: investigation. K.F.: investigation. T.M.: supervision. Z.S.: conceptualization, methodology, supervision, writing—review and editing. All authors have approved the final draft submitted.

**Financial support:** This work was supported by the research grants of the National Research, Development and Innovation Office (grant IDs: 125377 and 143549 to TM, 129266 to AB, and 134863 to FK), by the New National Excellence Program of the Ministry of Human Capacities (UNKP-23-3, SZTE-268, and EKÖP-368 to BP) and Janos Bolyai Research Grant (BO/00723/22 to BR). The funding body did not play roles in the design of the study and collection, analysis, and interpretation of data and in writing the manuscript.

**Potential competing interests:** The authors declare to have no competing interests.

**Study Highlights**

**WHAT IS KNOWN**

✓ The COVID-19 pandemic substantially disrupted gastroenterology care and patient's healthcare access.
✓ Colorectal cancer (CRC) screening favorably influences epidemiological outcomes.

**WHAT IS NEW HERE**

✓ Population-based CRC screening only partly offset the negative impact of COVID-19 on CRC epidemiology during the pandemic but led to favorable postpandemic shifts in clinical presentation.
✓ Noninvasive prescreening methods, such as fecal immunochemical test within population-based screening programs, may mitigate the adverse epidemiological impact of the COVID-19 pandemic on CRC.

## ABBREVIATIONS:

AJCC: American Joint Committee on Cancer
CRC: colorectal cancer
ESGE: European Society of Gastrointestinal Endoscopy
FIT: fecal immunochemical test
ICD: International Classification of Diseases
NNGYK: Hungarian National Centre for Public Health and Pharmacy
PCR: Polymerase Chain Reaction
STROBE: Strengthening the Reporting of Observational Studies in Epidemiology
SVI: Social Vulnerability Index
TNM stage: Tumor, Node, Metastasis stage
WEO: World Endoscopy Organization
